# Cultural Adaptation, Validation, and Analysis of the Self-Efficacy in Palliative Care Scale for Use with Spanish Nurses

**DOI:** 10.3390/ijerph16234840

**Published:** 2019-12-02

**Authors:** Raquel Herrero-Hahn, Rafael Montoya-Juárez, César Hueso-Montoro, Celia Martí-García, Diego Alejandro Salazar-Blandón, María Paz García-Caro

**Affiliations:** 1 University Health Provider Institution, University of Antioquia, Medellín 050010, Colombia; raquelherrero@ugr.es; 2 Faculty of Health Sciences, Psychosocial and Transcultural Aspects of Health and Illness (CTS436) Research Group, University of Granada, 18071 Granada, Spain; 3 Faculty of Health Sciences, Psychosocial and Transcultural Aspects of Health and Illness (CTS436) Research Group, University of Malaga, 29071 Malaga, Spain; 4 Faculty of Nursing, University of Antioquia, Medellín 050010, Colombia

**Keywords:** validation studies, nursing, nursing students, palliative care, self-efficacy

## Abstract

The aim of the present study is to validate the Self-Efficacy in Palliative Care Scale (SEPC) in Spanish nursing professionals and students, to describe their levels of self-efficacy, and to determine the influencing factors. A validation study and a cross-sectional descriptive study were carried out, with the data analysed using contrast tests and multiple linear regression; 552 nurses and 440 nursing students participated. The Spanish version consists of 23 items and has a high degree of reliability (α = 0.944). Confirmatory factor analysis revealed one additional factor (i.e., management of psychosocial and spiritual aspects) in comparison to the original scale. Contrast tests revealed that the mean SEPC score was higher in professionals than in students (*p* < 0.001) and that the professionals who had higher levels of self-efficacy were older (*p* < 0.001), had more previous training (*p* < 0.001), and had more experience in end-of-life care (*p* = 0.001). The linear analysis results confirm a significant association between age and previous training in end-of-life care. The Spanish version of the SEPC is a reliable tool for both nursing professionals and students. The level of self-efficacy of both groups is moderate and is influenced by age, experience, and training in end-of-life care.

## 1. Introduction

Many chronically ill patients will develop palliative care needs towards the end of their lives [1]. Health professionals need to acquire the competencies to respond adequately to these palliative care needs in both patients and their families [2]. Nurses play a crucial role in this regard, but the training they receive in palliative care has been shown to be insufficient [3].

Training in these competencies may improve nursing professionals’ attitudes toward end-of-life care [4], their levels of anxiety [5], and even the decision-making skills of patients and their families [6].

Conversely, the lack of training in these competencies may lead to stress and anxiety in professionals when caring for end-of-life patients, which may affect, in the long term, their own health [7,8,9,10].

Bandura [11] defines competence as the ability to translate secondary skills (cognitive, social, emotional, and sensorimotor), knowledge, values, and attitudes into appropriate actions. Self-efficacy, in turn, is an individual’s assessment of their ability to organise and implement the courses of action required to achieve the designated objectives [12]. Self-efficacy therefore influences the acquisition, development, and attainment of competencies. Individuals with a high level of professional self-efficacy set higher goals for themselves and persist when faced with difficulties, which they consider to be challenges rather than threats [13].

Self-efficacy is a useful concept in research: It has been included, for example, as a dimension of the Healthcare Professional Humanization Scale (HUMAS) [14] and has been related to quality of life in patients with metabolic complications [15].

High-quality palliative care requires both the competence and self-efficacy of professionals [16]. For this reason, it is important to have tools available that make it possible to assess how confident health professionals feel in their ability to provide adequate care to these patients.

One of the tools used to assess practitioners’ self-efficacy in relation to end-of-life care is the Self-Efficacy in Palliative Care Scale (SEPC). The SEPC was developed by Mason and Ellershaw [17] in the United Kingdom based on a previous questionnaire assessing the effect of a palliative care training programme on Australian medical students [18].

This scale is based on the theoretical principles of Bandura’s Social Cognitive Theory and consists of 23 items that assess perceived efficacy in relation to communication (8 items), patient management (8 items), and multiprofessional teamworking (7 items). Each subscale presents a set of specific behaviours and skills in which the perceived efficacy of successfully performing each behaviour or skill is assessed using a 100-mm visual analogue scale ranging from “very anxious” to “very confident.” The reliability and validity of the scale were determined with medical students, yielding a Cronbach’s α value greater than 0.92 on all subscales. Exploratory factor analysis revealed 3 factors that confirmed the item distribution in the three subscales and explained more than 68% of the variance [17].

The SEPC was used in a multicentre pilot study aimed at ascertaining the degree of preparedness in palliative care of newly graduated medical students in five European countries (France, Ireland, Italy, Spain, and the United Kingdom). Versions of the scale translated into the language of each country were used, although they had not been validated [19]. In Canada, in a study that sought to assess the confidence that medical students and internal medicine residents had in different aspects of palliative care, the SEPC communication subscale was used without prior psychometric validation analysis [20]. Recently, Rai and Mason [21] transposed the SEPC to an electronic format to integrate it in an electronic tool in order to assess the effect of undergraduate training in palliative care.

The SEPC has been used in a multicentre study in long-term care facilities in 6 European countries (Belgium, England, Finland, Italy, The Netherlands, and Poland) [22], but, although a forward–backward translation was conducted in each country, the authors did not report data about formal validation except in the original English version. This scale has also been used in a study to identify determinants of high self-efficacy in nurses and care assistants of long-term-care centres in Germany, but the researchers did not specify if they used a translated or a validated German version [23].

The translation of a measuring instrument, as a process, is necessary but not in itself sufficient to ensure its validity in a different setting to that for which it was designed [24]. The objectives of the present study were, therefore, the following: to realise the cultural adaptation and validation of the SEPC scale in Spanish nursing professionals and students by assessing its face validity, content validity, construct validity, reliability, and feasibility and to determine the level of self-efficacy in palliative care of students and professionals, as well as the influences that sex, age, professional experience, and previous training and experience in palliative care all have on self-efficacy.

## 2. Materials and Methods

### 2.1. Design

A cultural adaptation and scale validation study was carried out following the process outlined by Ramada, Serra, and Delclós [25]. This process includes (a) the cultural adaptation of the instrument, which consists of the translation, back-translation, review by a committee of experts, and pilot testing of the scale, and (b) the validation of the instrument, through the verification of the psychometric properties that determine its reliability, validity, and feasibility. Subsequently, a quantitative study was carried out with a descriptive-analytical and cross-sectional design to assess the influence of related variables.

### 2.2. Cultural Adaptation

With the permission of the author of the original scale, the SEPC was translated from English into Spanish by two independent native Spanish translators. Subsequently, a synthesis of the translations was made and submitted in two rounds to a committee of experts (CE) made up of twelve professionals, with at least 5 years’ clinical and/or research experience in palliative care, for review and evaluation of the semantic equivalence, content validity, face validity, and feasibility of the instrument using a Likert scale. The minimum level of agreement among experts was set at 80% [26]. In addition, the experts were asked to make comments and suggestions for the adaptation of the items, so that they would be understandable and applicable in the Spanish context. Once the recommendations made by the CE had been consolidated and agreement had been reached among the experts, the preliminary version obtained was back-translated by two bilingual native English translators and compared with the original version.

With this preliminary version, the pilot test was carried out in person with a group of 40 professionals who had similar characteristics to the population under study. These individuals were subsequently excluded from participating in the main study [20]. They were provided with the SEPC scale, a sociodemographic information form, and another form to report their comments on the scale. This pilot test served to establish whether the questionnaire could be satisfactorily understood and completed by all professionals and to estimate the completion time required. It had been established that items that were reported as difficult to understand by 15% or more of the participants would be modified to refine the final version of the instrument [21]. However, no item met this criterion and no further modifications were necessary.

### 2.3. Setting and Sample

The population under study consisted of Spanish nurses registered in their corresponding professional associations, as well as undergraduate nursing students.

Intentional sampling was used, seeking the participation of Spanish nurses who were currently working in Andalusia, who had one year or more of professional experience, whose mother tongue was Spanish, and who had an e-mail address registered in the database of their corresponding professional associations. In addition, fourth-year nursing students were recruited from Andalusian public universities. Data were collected between January 2017 and March 2018.

Table 1 shows the sociodemographic characteristics of the participants. A total of 992 individuals participated in the study, with 440 nursing students and 552 nursing professionals. According to the National Institute for Statistics (INE), in 2016 (last year recorded) [27], there was a population of 41.060 registered nurses in Andalusia, while there were 2040 nursing students in Andalusian public universities in their fourth year of study in 2017–2018.

Among the students, 85.91% was female and the mean age was 21.97 years old (SD ± 4.569, 95% CI: 21.54–22.40). Of the professionals, 80.07% was female and the mean age was 37.08 years old (SD ± 12.696, 95% CI: 36.02–38.14); 12.32% of the professionals reported having had specialty training and 28.44% reported having postgraduate education. Of the students and professionals, respectively 40.68% and 61.05% reported having had previous training in palliative care or end-of-life care. On the other hand, 22.73% of the students and 60.14% of the professionals reported having previous professional and/or personal experience related to end-of-life care.

### 2.4. Instruments and Data Collection

In conjunction with the Spanish version of the Self-Efficacy in Palliative Care Scale (SEPC-S), a sociodemographic information form was administered to obtain data such as age, sex, professional experience, level of education, and previous end-of-life training and experience.

The SEPC-S, together with the sociodemographic information form and the informed consent form, were entered into a free-access virtual platform for administering online questionnaires. The URL of this platform was e-mailed to professionals through professional associations as well as to the fourth-year nursing faculty, so that it could be passed on to students.

### 2.5. Data Analysis

Data analysis was carried out using IBM-SPSS statistical software version 22 for Windows (SPSS Inc., Chicago, IL, USA) and FACTOR 10.8.03 (freeware program developed at the Rovira i Virgili University, Tarragona, Spain). Descriptive statistics were used to profile the sample, and various types of analysis were used to assess the psychometric aspects of the instrument. Face validity and semantic equivalence were determined based on the results obtained from the review by the CE and the pilot test. Content validity was determined based on the assessments conducted by the CE using Lawshe’s Modified Content Validity Index (CVI) [28].

Reliability was determined by assessing internal consistency by calculating Cronbach’s *α* for the whole scale and for each subscale and by assessing changes in the subscales caused by the removal of each item from the subscales. To determine construct validity, the Kaiser–Meyer–Olkin (KMO) test and Bartlett’s test of sphericity were used to confirm whether it was possible to perform subsequent factor analyses with the scale. The KMO test and Bartlett’s test of sphericity returned satisfactory results (KMO = 0.919; *χ*^2^ = 9863.89; *p* < 0.001).

The data were divided into 3 groups for analysis. The first group was made up of all of the individuals and the remaining two groups were made up of students or professionals only (STU&PROF, STU, and PROF, respectively). As no evidence was found in favour of the assumption of multivariate or univariate normality in either group, the unweighted least squares method was used to estimate the parameters in the confirmatory factor analysis [29]. The model was run with the original 3-factor structure, and goodness-of-fit indices were calculated, revealing a poor fit of the model and, in the group of professionals, the transfer of three items to a new factor. As a result, a new 4-factor model was explored in all cases.

The time required to complete the scale, the simplicity of the format, and the clarity of the items were evaluated to determine the feasibility of the scale.

In order to assess the influence of self-efficacy related variables, nonparametric tests were used (the Mann–Whitney *U*-test and Spearman’s rho), given the heterogeneity found when conducting normality tests (Kolmogorov–Smirnov and Shapiro–Wilk tests). Additionally, a multiple linear regression analysis was conducted, with the SEPC-S score being the dependent variable, and the variables which presented significant differences in bivariate analyses were used as independent variables (age, sex, and previous experience and training in end-of-life care). Linearity between the dependent variable and age was confirmed by plotting aggregate variables, and the absence of collinearity between independent variables was verified, considering a variance inflation factor <2.5 as valid. Homoscedasticity was confirmed with the Breusch–Pagan test (*p* > 0.05); and the normality of the error distribution was verified using the Shapiro–Wilk test (*p* > 0.05). R commander (R version 3.2.2, https://www.r-project.org/, Spanish R-UCA Project, http://knuth.uca.es/R) was used for these analyses.

### 2.6. Ethical Considerations

The present study complies with the basic ethical principles for the responsible conduct of research involving people. Informed consent was requested from all participants, and authorisation was obtained from the author of the scale, Stephen Mason, to carry out the cultural adaptation and validation of the scale in the Spanish context. Approval was obtained from the Research Ethics Committee of the University of Granada, Spain, as per record no. 270/CEIH/2017.

## 3. Results

### 3.1. Linguistic Validation

The process of cultural adaptation of the scale to the Spanish context took place between June and October 2016. Adjustments were made in accordance with the recommendations made by the CE by changing some terms to suit the context in which the scale was to be used. The number of items was not modified, but the response format was. As a result, the visual analogue scale was replaced by a Likert scale ranging from 1 to 10 in order to facilitate the evaluation of the SEPC-S.

These changes made it possible to determine semantic equivalence and content validity (CVI: 0.967). There was agreement that the scale appeared to measure what it was intended to measure (87.50%), including aspects related to the construct of self-efficacy in palliative care. The items were relevant and easy to understand. Appendix A shows the modifications made to the terms and wording of the items.

### 3.2. Feasibility

The time required to complete the scale, the simplicity of the format, and the clarity of the items were assessed to determine the feasibility of the scale. In the pilot test, the mean amount of time required to complete the scale was 3.68 min (SD ± 2.080), which was considered to be an appropriate amount of time by 92.50% of the participants. According to the comments made by the participants, all items were of interest to 97.50% of the participants, all items were easily understood by 92.50% of the participants, and 97.50% of the participants indicated that the SEPC-S had a simple format.

### 3.3. Construct Validity

In the group of students and professionals together and in the group of students only, the structure suggested by the author is observed, in which items 1 to 8 are related to communication, items 9 to 16 are related to patient management, and items 17 to 23 are related to multiprofessional teamworking. However, in the group of professionals only, items 14–16 were more related to the communication factor than to patient management. In all cases, a poor fit of the 3-factor model was obtained (RMSEA > 0.05).

When considering a 4-factor model, it can be observed in Table 2 that, in all groups, items 14–16 migrate to the new factor, with correlations greater than 0.50; the communication and multiprofessional teamworking factors do not vary. This distribution divides the patient management subscale into two subscales. The first subscale, labelled patient management—physical, consists of items 9–13 and is related to the management of the patients’ physical aspects. The second subscale, labelled patient management—psychosocial-spiritual, consists of items 14–16 and is related to the management of psychological, social, and spiritual aspects of patients. This 4-factor model presents a better fit in all indicators and in all groups (Table 3). For the combined group of students and professionals, although the value for the RMSEA dropped from 0.077 to 0.055, this is not considered to be a good fit, as the RMSEA is expected to be less than 0.05 [30].

The 4-factor model fits best when applied to both groups independently. Therefore, the rest of the analyses have been made on the basis of this model, considering nursing students and professionals independently.

### 3.4. Reliability

Cronbach’s *α* value was greater than 0.944 for the SEPC-S in both groups. For each subscale, the Cronbach’s *α* value was between 0.895 and 0.952. There was no item of which the removal caused the value of Cronbach’s *α* to increase.

### 3.5. Level of Self-Efficacy in Palliative Care: Association and Correlation between Variables

The SEPC-S mean scores were 6.53 (SD ± 1.389) in students and 6.91 (SD ± 1.298) in professionals. The mean scores of the communication subscale were 6.26 (SD ± 1.634) and 6.66 (SD ± 1.600) for students and professionals, respectively. The mean scores for the perceived level of confidence in the management of patients’ physical aspects and in the management of patients’ psychological, social, and spiritual aspects were 6.36 (SD ± 1.793) and 6.12 (SD ± 2.003) in students, respectively, and 7.23 (SD ± 1.391) and 6.30 (SD ± 1.819) in professionals, respectively. The mean scores of the teamworking subscale were 7.14 (SD ± 1.805) in students and 7.22 (SD ± 1.624) in professionals.

When comparing the results obtained from students with those obtained from professionals, statistically significant differences (*p* < 0.001) were found in the level of self-efficacy in palliative care, in the communication subscale, and in the subscale regarding the management of patients’ physical aspects between students and professionals. However, these differences were not significant in the subscale concerning the management of psychological, social, and spiritual aspects of patients (*p* = 200) or in the teamworking subscale (*p* = 0.866) (Table 4).

When comparing the results obtained on the basis of sex, statistically significant differences were found (*p* < 0.05) in the level of self-efficacy in communication between men and women in students and professionals, with these levels of confidence being higher in men.

In addition, statistically significant differences (*p* < 0.05) were found in the level of self-efficacy in palliative care and in all its dimensions between professionals who had previous training in end-of-life care and those who did not and in the level of self-efficacy in palliative care in the subscales of communication and management of the physical aspects of patients between professionals who had previous experience in end-of-life care and those who did not, with a higher level of self-efficacy being found in professionals who had previous training or experience in this field (Table 5). No significant differences were found between professionals who had specialty training versus those who did not (*p* > 0.525) or between professionals who had postgraduate education and those who did not (*p* > 0.461).

With respect to age, positive correlations were found between age and self-efficacy in communication in students (*ρ* = 0.110; *p* = 0.021); as well as between age and self-efficacy in palliative care (*ρ* = 0.231), communication (*ρ* = 0.285), patient management—physical (*ρ* = 0.150), and patient management—psychosocial-spiritual (*ρ* = 0.272) in professionals (*p* < 0.001). Similarly, there were positive correlations between years of professional experience and self-efficacy in palliative care (*ρ* = 0.225); communication (*ρ* = 0.268); management of physical aspects (*ρ* = 0.170); and management of psychological, social, and spiritual aspects (*ρ* = 0.249) (*p* < 001).

Finally, the results of the linear regression analysis shown in Table 6 confirm a significant association between age and prior training in end-of-life care.

## 4. Discussion

In the present study, the methodological process for the cultural adaptation and validation of the SEPC in nursing students and professionals in Spain was carried out. The Spanish version presented an adequate factorial structure and high internal consistency. There were statistically significant differences in scale scores according to sex in students and according to sex, age, professional experience, and previous experience and training in palliative care in professionals.

The adaptation of the SEPC to the Spanish context required the modification of some items. The term cancer was replaced by illness so that terminally ill patients were not excluded because of having a non-oncological illness. The term pain was also replaced by symptoms, so that the management of the patients’ physical aspects did not refer solely to pain. These changes are in line with the palliative care goals set by the World Health Organization [31]. Additionally, referral for psychiatric evaluation was changed for referral for psychological evaluation and referral to a lymphoedema service was changed for referral to an advanced palliative care service, as this is more common in the Spanish end-of-life care context. These modifications were made on the basis of the experts’ recommendations and made it possible to obtain a CVI close to 1.

The SEPC version adapted to the Spanish context was shown to have a high internal consistency, similar to the consistency of the original scale [17]. However, the factor structure found differs from the structure reported in the original validation study. In this study, it was found that the best fitting model, using the unweighted least squares method, is made up of 4 factors. In this model, the communication subscale and the multiprofessional teamworking subscale are, however, the same as in the original scale [17]. It is only the patient management subscale which changes, being divided into two subscales: a first subscale made up of the 5 items referring to the management of physical aspects and another subscale made up of the 3 items relating to the management of psychological, social, and spiritual aspects. The differentiation made by the patient management subscale between physical aspects and psychosocial-spiritual aspects could be explained by the fact that, although there seems to be a paradigm shift towards a patient-centred approach, the general focus of nursing care remains on physical aspects while psychosocial aspects are addressed as secondary [32,33,34,35].

The 4-factor model fits best when applied to the two populations (students and professionals) independently, so it is recommended that future studies use the SEPC-S conduct analyses in this way.

For self-efficacy in palliative care, students obtained a score of 6.53 and professionals obtained a score of 6.91, both scores indicating a moderate level on a scale of 1 to 10. These scores are similar to those found in previous studies using the same scale in medical students [36].

The lack of preparedness of students and professionals is a common feature in the scientific literature. In a multicentre study published in 2017 with Intensive Care Units doctors in Germany, 67.6% of the professionals stated that they felt little or no confidence in tackling basic aspects of palliative care [37]. According to Winthereik et al. [38], 76.1% of general practitioners in Denmark reported that they felt confident in treating end-of-life patients. However, this varied substantially depending on the aspect to be treated (56–89%).

Regarding the factors that may have influenced this score, the results of this study show significant differences in the communication subscale according to sex, finding a higher level of self-efficacy in men. The results found in the literature regarding differences in self-efficacy between men and women vary greatly. Authors who have studied the levels of general self-efficacy and self-efficacy in relation to different aspects reported a higher level of general self-efficacy in men [39,40,41]. Other studies found no difference between men and women in levels of self-efficacy or reported differences based on the aspect to which self-efficacy referred [42,43]. A study conducted among university students in Mexico [44] concluded that, although men reported better self-efficacy in problem solving than women, women reported higher levels of communication self-efficacy than men. In a similar context, a study pointed out that women perceived themselves as more self-efficient in academic task related to communication than men do [45].

Although a recent review highlighted that communication and language skills development is faster and more advanced in women compared with men [46], different studies indicate that the differences in self-efficacy between men and women do not only stem from sex-based differences but from gender-based differences, since expectations, rules, and norms are generated for each individual based on the meanings that are culturally attributed to belonging biologically to one sex or the other, which is in turn associated with a higher level of self-efficacy for the specific tasks of the assigned gender role [47].

An association was also found between the professionals’ level of self-efficacy in palliative care and their previous training in end-of-life care, both in contrast tests and in the linear regression model, with a higher level being observed in professionals who had received training. This is consistent with previous studies, which point to training as a key aspect [22,23,36,37,48]. One study in the United Kingdom evaluated the change in scale scores in medical students after a theoretical-practical training programme in palliative care and observed an increase in the subscales of communication, patient management, and teamworking [36]. Subsequently, a study was carried out with the aim of determining whether training time influenced the degree of improvement in the level of self-efficacy. Two groups of medical students were studied. The first group received 8 days of theoretical-practical training in palliative care, whereas the second group received 16 days of training. A significantly greater improvement in the level of self-efficacy in palliative care was found in the group that received more days of training [37]. Other studies found that, after an end-of-life care training programme, the level of self-efficacy in doctors and nurses increased [22,49,50]. Although they did not use the same instrument, Phillips, Salamonson, and Davidson [48] reported improved self-efficacy in Australian nurses and nursing assistants after following a training programme.

Although the World Health Organization has been urging governments for years to integrate palliative care training into undergraduate, postgraduate, and continuing education [51], data from the recently published Atlas of Palliative Care in Europe [52] indicate that, in 16 countries of the European Union, there is no nursing faculty where a specific compulsory palliative care subject is taught and that only in 4 countries is this subject compulsory for all nurses. In Spain, the government advocates that training in palliative care be initiated in nursing undergraduate studies and be complemented by continuing and postgraduate education [53]. However, in 2013, only 49.10% of Spanish universities included palliative care as a compulsory subject [54]. The results of this study highlight the need to expand training in palliative care to all training centres.

Furthermore, an association was found between the professionals’ level of self-efficacy in palliative care and their previous experience in end-of-life care, with a higher level being observed in professionals who reported having previous experience. However, although the regression analysis also shows the existence of an association, this did not reach statistical significance. This may be explained by the fact that the type of experience and duration were not taken into account, which could affect the results.

This relationship is also not very clear in previous studies. End-of-life experience is not usually included in studies that evaluate the effectiveness of educational interventions, and there is no consensus on how this experience should be included in these studies [55]. For a palliative care training programme, Kirkpatrick, Cantrell, and Smeltzer [56] established two intervention groups based on whether the subjects had experience in end-of-life care. Although both groups improved their competence in palliative care, the authors found no significant differences based on previous experience.

In this sense, Benner, Tanner, and Chesla [57] argue that experience-based skills acquisition is safer and faster when it takes place on a solid educational foundation. Applying the acquired knowledge to the resolution of different practical problems allows the student to gradually acquire skills and abilities [57,58]. In the aforementioned study by Reed et al. [49], professionals place special emphasis on the fact that the direct and immediate application of the knowledge acquired in day-to-day training is the key to improving clinical practice. This means that theoretical knowledge as well as experience in direct contact with patients and families must be effectively integrated into nursing training.

Finally, a correlation was found between age and duration of professional experience and the level of self-efficacy. This was to be expected, since older professionals are more likely to have more professional experience and experience in end-of-life care, thus improving self-efficacy. Wilson, Avalos, and Dowling [59] pointed out that, as age and accumulated professional experience increase, knowledge of palliative care and attitudes towards dying patients improve. Similarly, in a study conducted in Taiwan [60], nurses and nursing assistants in nursing homes showed greater knowledge of the care they provided to patients with advanced dementia as their clinical experience increased.

Further studies might analyze the relationship between self-efficacy and other related concepts. In the Spanish context, one of the concepts that have been related to self-efficacy is attitude toward death and dying. In that sense, the Frommelt’s Attitude Toward Care of the Dying Scale (FATCOD) correlates, as does the SEPC, with palliative care training and experience [61]. Bugen’s Coping with Death Scale has also been used as a measure of self-competence in nursing, physiotherapy, and medicine undergraduate students [62].

Due to limitations in access to the sample, the study design did not support the random sampling of subjects. As previously mentioned, although the end-of-life experience variable was taken into consideration, the type of experience and duration were not, which could affect the results. Due to the heterogeneity found when performing the normality tests, nonparametric tests were used in the analyses.

## 5. Conclusions

The cultural adaptation and validation of the SEPC for use in Spain has resulted in a valid instrument for determining the nursing students’ and professionals’ levels of self-efficacy in palliative care. The 4-factor model obtained fits adequately with students and professionals and has a high internal consistency index, which demonstrates the validity and reliability of the instrument.

The level of self-efficacy in palliative care of nursing professionals and students in Andalusia, Spain, is moderate. On the other hand, the levels of self-efficacy in palliative care as well as self-efficacy in communication and patient management are higher in professionals than in students. Self-efficacy in communication is greater in men than in women both in students and in professionals.

The level of self-efficacy in palliative care is higher in professionals who have previous training and/or experience in end-of-life care. This relationship also applies to older professionals and more experienced professionals. Given their influence on self-efficacy in palliative care, training and previous experience can be viewed as opportunities to improve both the confidence that professionals feel when caring for patients at the end of their lives and the quality of care.

## Figures and Tables

**Table 1:** Sociodemographic characteristics of the participants (*n* = 992).

	Students		Professionals	
Variables	*n* (%)	Mean (SD)	*n* (%)	Mean (SD)
Age	440 (100)	21.97 (±4.569)	552 (100)	37.08 (±12.696)
Sex				
Female	378 (85.91)		442 (80.07)	
Male	62 (14.09)		110 (19.93)	
Professional experience (years)				12.88 (±12.44)
Specialty training				
Yes			68 (12.32)	
No			484 (87.68)	
Postgraduate education				
Yes			157 (28.44)	
No			395 (71.56)	
Previous training in end-of-life care				
Yes	179 (40.68)		337 (61.05)	
No	261 (59.32)		215 (38.95)	
Previous experience in end-of-life care				
Yes	100 (22.73)		332 (60.14)	
No	340 (77.27)		220 (39.86)	

Source: Sociodemographic information form completed by Spanish nursing students and professionals.

**Table 2:** Factor loadings of a 4-factor model of the Self-Efficacy in Palliative Care Scale Spanish version (SEPC-S) in nursing students and professionals.

Variable ^1^	STU&PROF ^2^				STU ^3^				PROF ^4^			
	F 1	F 2	F 3	F 4	F 1	F 2	F 3	F 4	F 1	F 2	F 3	F 4
**SEPC_1**		0.66				0.69				0.63		
**SEPC_2**		0.64				0.63				0.64		
**SEPC_3**		0.82				0.83				0.82		
**SEPC_4**		0.84				0.85				0.83		
**SEPC_5**		0.82				0.85				0.78		
**SEPC_6**		0.69				0.70				0.66		
**SEPC_7**		0.65				0.69				0.60		
**SEPC_8**		0.61				0.62				0.57		
**SEPC_9**			0.55				0.54				0.54	
**SEPC_10**			0.83				0.84				0.79	
**SEPC_11**			0.82				0.84				0.78	
**SEPC_12**			0.83				0.82				0.83	
**SEPC_13**			0.83				0.83				0.81	
**SEPC_14**				0.65				0.62				0.64
**SEPC_15**				0.69				0.61				0.70
**SEPC_16**				0.74				0.70				0.77
**SEPC_17**	0.59				0.66				0.53			
**SEPC_18**	0.87				0.88				0.85			
**SEPC_19**	0.90				0.89				0.90			
**SEPC_20**	0.87				0.89				0.84			
**SEPC_21**	0.71				0.83				0.62			
**SEPC_22**	0.77				0.83				0.72			
**SEPC_23**	0.57				0.63				0.54			

^1^ Unweighted least squares extraction method with varimax rotation. Only the highest coefficient for each factor is presented. ^2^ Students and professionals. ^3^ Students. ^4^ Professionals.

**Table 3:** Goodness and fit indices for the SEPC-S factorial structure models in nursing students and professionals.

Model ^1^	*χ* ^2^	df	CFI	RMSR (95% CI)	RMSEA (95% CI)
**STU&PROF** 3	540.89	187	0.979	0.046 (0.042 0.050)	0.077 (0.042 0.084)
**STU** 3	229.69	187	0.988	0.045 (0.038 0.049)	0.059 (0.033 0.068)
**PROF** 3	313.87	187	0.986	0.047 (0.040 0.051)	0.064 (0.045 0.072)
**STU&PROF** 4	239.06	167	0.99	0.030 (0.028 0.032)	0.055 (0.023 0.060)
**STU** 4	129.05	167	0.996	0.034 (0.030 0.037)	0.034 (0.007 0.048)
**PROF** 4	131.03	167	0.997	0.030 (0.028 0.032)	0.030 (0.008 0.034)

^1^ Minimum fit function chi-square (*χ*^2^); degrees of freedom (df); comparative fit index (CFI); root mean square of residuals (RMSR); and root mean square error of approximation (RMSEA).

**Table 4:** The participants’ level of self-efficacy in palliative care and differences between students and professionals (*n* = 992).

	Students		Professionals		
Variables	Mean (SD)	95% CI	Mean (SD)	95% CI	*p* ^1^
SEPC-S	6.53 (±1.389)	6.40–6.66	6.91 (±1.298)	6.80–7.01	<0.001
Communication	6.26 (±1.634)	6.10–6.41	6.66 (±1.600)	6.52–6.79	<0.001
Patient management—physical	6.36 (±1.793)	6.20–6.53	7.23 (±1.391)	7.11–7.35	<0.001
Patient management—psychosocial-spiritual	6.12 (±2.003)	5.93–6.31	6.30 (±1.819)	6.15–6.46	0.200
Multiprofessional teamworking	7.14 (±1.805)	6.97–7.30	7.22 (±1.624)	7.08–7.35	0.866

^1^ Mann–Whitney *U*.

**Table 5:** Association between sex, previous training and experience in end-of-life care, and the different dependent variables.

	Sex	Mid-Range	*p* ^1^	Previous Training in End-of-Life Care	Mid-Range	*p* ^1^	Previous Experience in End-of-Life Care	Mid-Range	*p* ^1^
Students									
SEPC-S	Male	238.77	0.222	Yes	224.46	0.588	Yes	233.86	0.232
	Female	217.50		No	217.78		No	216.57	
Communication	Male	253.21	0.029	Yes	221.51	0.890	Yes	241.10	0.065
	Female	215.13		No	219.81		No	214.44	
Patient management—physical	Male	232.67	0.416	Yes	230.15	0.187	Yes	222.96	0.826
	Female	218.50		No	213.88		No	219.78	
Patient management—psychosocial-spiritual	Male	241.47	0.161	Yes	215.44	0.489	Yes	223.42	0.794
	Female	217.06		No	223.97		No	219.64	
Multiprofessional teamworking	Male	210.21	0.492	Yes	217.44	0.676	Yes	226.48	0.592
	Female	222.19		No	222.60		No	218.74	
Professionals									
SEPC-S	Male	296.32	0.145	Yes	296.47	<0.001	Yes	294.75	0.001
	Female	271.57		No	245.19		No	248.96	
Communication	Male	304.50	0.040	Yes	296.45	<0.001	Yes	299.74	<0.001
	Female	269.53		No	245.23		No	241.43	
Patient management—physical	Male	293.52	0.210	Yes	293.96	0.001	Yes	296.52	<0.001
	Female	272.26		No	249.13		No	246.29	
Patient management—psychosocial-spiritual	Male	277.75	0.926	Yes	289.82	0.014	Yes	286.27	0.077
	Female	276.19		No	255.62		No	261.76	
Multiprofessional teamworking	Male	291.68	0.264	Yes	290.90	0.008	Yes	281.24	0.391
	Female	272.72		No	253.93		No	269.35	

^1^ Mann–Whitney *U*.

**Table 6:** Multiple linear regression.

Model ^1^	Coefficient	Standard Error	*t*	*p*	VIF *
**Constant**	6.477	0.201	32.180	<0.001	
**Age**	0.023	0.004	5.515	<0.001	1.012
**Sex**					1.001
Male	Reference				
Female	−0.243	0.130	−1.868	0.062	
**Previous experience in end-of-life care**					1.189
Yes	Reference				
No	−0.210	0.115	−1.820	0.069	
**Previous training in end-of-life care**					1.175
Yes	Reference				
No	−0.327	0.116	−2.833	0.005	

^1^ Summary of the model and adjustment conditions: F = 13.95; standard error = 1.217; *p* < 0.001; R ^2^ = 0.093; adjusted R ^2^ = 0.09; linearity of quantitative independent variables was verified by plotting aggregate variables; absence of collinearity was verified by Variance Inflation Factor (VIF); normality of errors: Shapiro–Wilk test with *p* = 0.065; homoscedasticity: Breusch–Pagan test with *p* = 0.129. Decision rule for α = 0.10, 0.05 < *p* < 0.15 indicates that there are signs of significance and that the sample should be increased and the tests should be repeated.

## Appendix A

**Table A1:** Semantic analysis modifications.

Item	Original SEPC Item	Original Translation into Spanish from Spain	Version as Modified by the Committee of Experts and the Pilot Test
1	Discussing the likely effects of cancer with the patient	Hablar sobre los efectos previsibles del cáncer con el paciente	Hablar sobre los efectos probables de la enfermedad con el paciente
2	Discussing the likely effects of cancer with the patient’s family	Hablar sobre los efectos previsibles del cáncer con la familia del paciente	Hablar sobre los efectos probables de la enfermedad con la familia del paciente
4	Discussing the patient’s own death (with the patient)	Hablar de la muerte del propio paciente (con el paciente)	Hablar con el paciente sobre su propia muerte y las decisiones relacionadas con la misma
5	Discussing the patient’s death (to occur) with the family	Hablar sobre la muerte del paciente (próxima a ocurrir) con la familia	Hablar con la familia sobre la proximidad de la muerte del paciente y las decisiones relacionadas con la misma
12	Your ability to prescribe appropriate and adequate pain control medication	Su capacidad para prescribir los medicamentos adecuados y suficientes para el control del dolor	Su conocimiento de los medicamentos adecuados y suficientes para el control de síntomas
13	Your knowledge of the therapeutic and side-effects of analgesic agents	Su conocimiento de los efectos terapéuticos y secundarios de los analgésicos	Su conocimiento de los efectos terapéuticos y secundarios de los medicamentos para el control de síntomas
21	Appropriately referring palliative care patients to a lymphoedema service	Remitir, cuando proceda, a los pacientes de cuidados paliativos a un servicio de linfedema	Remitir, cuando proceda, a los pacientes en fase paliativa a un servicio avanzado de cuidados paliativos
22	Appropriately referring palliative care patients for psychiatric evaluation	Remitir, cuando proceda, a los pacientes de cuidados paliativos para una evaluación psiquiátrica	Remitir, cuando proceda, a los pacientes en fase paliativa o familiares para una evaluación psicológica
